# The Monitoring and Control of Task Sequences in Human and Non-Human Primates

**DOI:** 10.3389/fnsys.2015.00185

**Published:** 2016-01-21

**Authors:** Theresa M. Desrochers, Diana C. Burk, David Badre, David L. Sheinberg

**Affiliations:** ^1^Department of Cognitive, Linguistic and Psychological Sciences, Brown UniversityProvidence, RI, USA; ^2^Department of Neuroscience, Brown UniversityProvidence, RI, USA; ^3^Brown Institute for Brain Science, Brown UniversityProvidence, RI, USA

**Keywords:** sequential control, frontal cortex, monitoring, attention, executive functions, imaging studies, TMS, electrophysiology

## Abstract

Our ability to plan and execute a series of tasks leading to a desired goal requires remarkable coordination between sensory, motor, and decision-related systems. Prefrontal cortex (PFC) is thought to play a central role in this coordination, especially when actions must be assembled extemporaneously and cannot be programmed as a rote series of movements. A central component of this flexible behavior is the moment-by-moment allocation of working memory and attention. The ubiquity of sequence planning in our everyday lives belies the neural complexity that supports this capacity, and little is known about how frontal cortical regions orchestrate the monitoring and control of sequential behaviors. For example, it remains unclear if and how sensory cortical areas, which provide essential driving inputs for behavior, are modulated by the frontal cortex during these tasks. Here, we review what is known about moment-to-moment monitoring as it relates to visually guided, rule-driven behaviors that change over time. We highlight recent human work that shows how the rostrolateral prefrontal cortex (RLPFC) participates in monitoring during task sequences. Neurophysiological data from monkeys suggests that monitoring may be accomplished by neurons that respond to items within the sequence and may in turn influence the tuning properties of neurons in posterior sensory areas. Understanding the interplay between proceduralized or habitual acts and supervised control of sequences is key to our understanding of sequential task execution. A crucial bridge will be the use of experimental protocols that allow for the examination of the functional homology between monkeys and humans. We illustrate how task sequences may be parceled into components and examined experimentally, thereby opening future avenues of investigation into the neural basis of sequential monitoring and control.

## Introduction

We perform sequences of tasks every day. They range from the relatively simple and practiced, such as making a cup of coffee, to the more complex and infrequent such as cooking a three-course dinner for a large group of people. These sequences of tasks have common features. First, they are structured such that there is a superordinate goal that is served by multiple subordinate subgoals (Lashley, 1951). Second, the series of steps stay constant, but the specific sequences of motor actions can vary. Third, these sequences of tasks are often executed with little or no external cues as to the required order of the steps or which steps have already been completed.

**Figure 1:**
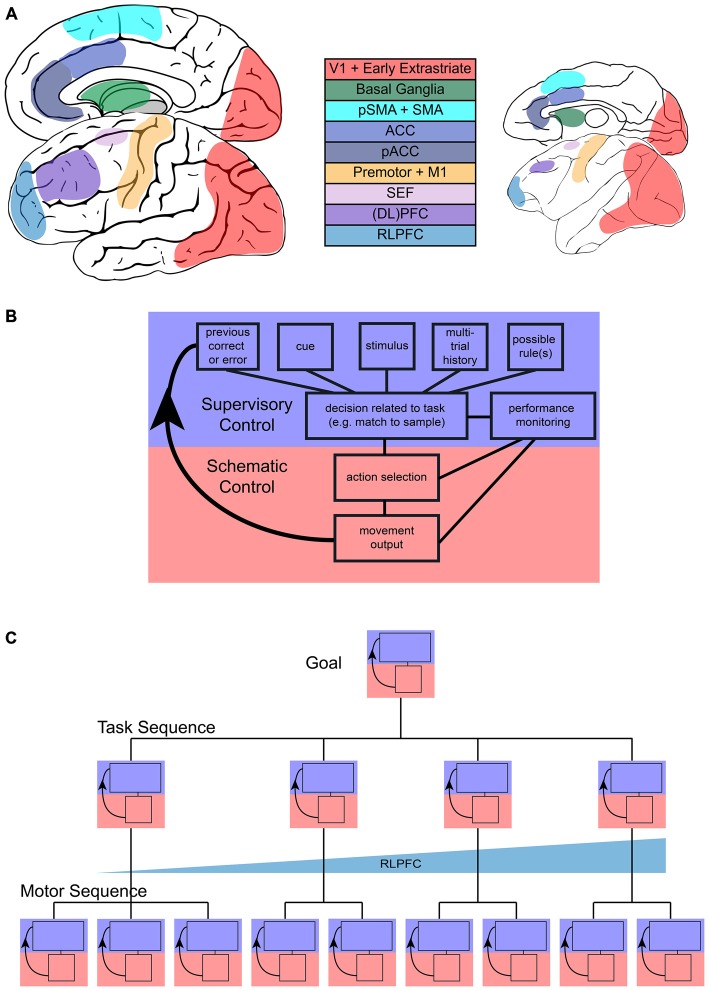
**Sequential task control schematic. (A)** Representation of main areas involved in sequential control in the human (left) and monkey (right) brain. **(B)** Flow of control in one step of a sequential task, with blue representing the increased involvement of supervisory control and red representing increased involvement of schematic control during a single step. **(C)** Representation of the multiple, hierarchical levels that can characterize sequences. Each step in more concrete motor sequences or more abstract task sequences may engage supervisory or schematic control and the interaction between them. Tracking across tasks in a task sequence may be accomplished by rostrolateral prefrontal cortex (RLPFC) ramping across multiple steps.

These common features hint at the underlying complexity of task sequences, and begin to illustrate the distinction between sequences that are automatic or proceduralized and sequences that require control in a more supervised manner. This distinction between automatic and supervised actions has been proposed before. Norman and Shallice (1986) contrasted a “contention scheduling” process that selected a series of habitual actions based on their value with a “supervisory attentional system.” The supervisory system was capable of overriding habitual action, but did not directly select individual actions. A similar distinction between two systems has also been made in the context of avoiding errors in action (Reason, 1990). There has been some disagreement as to the exact separation between these systems (e.g., see Botvinick and Plaut, 2004; Cooper and Shallice, 2006), but there is general agreement that a lapse in supervised control may lead to the automatic execution of a non-desired action. In this review, we will refer to the scheduler of the more automatic or proceduralized sequences of actions as the “schematic controller”, where schema are defined as sets of organized responses that can be executed as a unitary mass (Reason, 1990). The system that monitors, handles exceptions, and keeps track of progress towards a higher-level goal we will refer to as the “supervised controller”. These controllers are networks of areas (Figure 1A) that most likely function in feedback loops (Figure 1B). We will first address the kinds of sequences that typically fall under schematic control and then shift our main focus to the neural basis of sequences of cognitive tasks and their supervised control.

Although sequential tasks can seem simple because we execute them with relative ease, they require supervisory control. Anyone who has prepared coffee, but forgot to turn on the coffee maker in the morning, or has mistakenly put the can of peas in the refrigerator and the milk in the pantry has experienced a failure of this sequential task system. The kind of control necessary to execute sequences of tasks feels intuitively understood, and cognitive control functions have typically been attributed to the frontal cortex (Stuss and Benson, 1984; Miller and Cohen, 2001; Passingham and Rowe, 2002; Badre, 2008). However, specific deficits in sequential task execution have been difficult to pinpoint with classic clinical tests of cognitive function. Patients with frontal lobe dysfunction are impaired in their higher-order planning and sequencing capabilities and are not capable of independent living, yet they perform well on conventional tests of executive function (Eslinger and Damasio, 1985; Shallice and Burgess, 1991). Similarly, deficits in sequential multistep tasks are pervasive across many neurological and psychiatric disorders, and many patients cannot function normally in everyday life (e.g., Pauls et al., 2014). Thus, a better understanding of how we perform sequences of tasks and the underlying neural circuitry would make great strides towards helping large populations of people with deficits in these functions. In addition, we would contribute to our understanding of a fundamental, yet complex, daily behavior.

Investigation of sequential task performance must occur at multiple levels (Figure 1C) to understand both the high-level cognitive systems and the activity patterns at the neuronal level. This review examines what we know of the frontal and striatal neural circuits involved in motor sequences, monitoring, attention, and cognitive control that are all necessary in order to complete sequences of tasks. While we mainly present studies of visually guided tasks, we posit that task sequences driven by other modalities would use similar mechanisms. We also acknowledge that there is a rich literature encompassing the role of structures outside frontal and striatal circuitry, such as the hippocampus and medial temporal lobe (MTL), typically associated with navigation (e.g., Iglói et al., 2010; Pfeiffer and Foster, 2013) and learning/memory (e.g., Schendan et al., 2003; Ross et al., 2009; Albouy et al., 2013). Similarly, recent work has implicated the MTL in representing sequential patterns in stimuli (Schapiro et al., 2013, 2014; Wang et al., 2015). Although these systems almost undoubtedly interact with frontal and striatal systems and contribute to the performance of task sequences (for example reviews, see Ranganath and Ritchey, 2012; Dehaene et al., 2015), we do not focus on them here because they are outside the scope of our review. Schematic and supervisory control functions are not typically ascribed to the hippocampus and associated structures (McDonald and Hong, 2013).

Here, we integrate the findings of human and non-human primate studies in order to outline the interplay between schematic and supervised control circuits for the execution of task sequences. As the existing literature does not yet provide a comprehensive mechanism for sequential cognitive tasks, we assert the importance of investigating task sequences in human and non-human models. Furthermore, we propose a paradigm for the study of task sequences in non-human primates that would enable a direct investigation of the neural mechanisms underlying sequences of tasks. With this review, we aim to connect previous literature on schematic and supervised sequences, and motivate the field to pursue investigation of sequential task control.

## Motor Sequences

There is a rich literature examining the learning and execution of motor sequences, and the systems in the brain that support these sequences. While it is possible that these same systems are engaged in sequences of tasks, the extent to which this is true remains unknown. Further, an understanding of motor sequences is necessary, as many task sequences are composed of motor sequences. Many sophisticated behaviors, such as playing a musical instrument, require the concatenation of a series of complex motor acts. The series of steps can be preplanned and is often rehearsed to reduce variability and increase accuracy. Moreover, seemingly simple acts, such as reaching and grasping, also consist of multiple steps that rely more on subcortical areas and spinal cord circuits to appropriately execute (Whishaw et al., 2008; Azim et al., 2014). Here, we will discuss the literature that has investigated the neural basis of several kinds of motor sequences: muscle activation sequences, habitual motor sequences, and supervised movement sequences. Once learned, these sequences can be executed with minimal cognitive oversight and would fall under the purview of cognitive goals. Thus understanding the neural circuitry that underlies motor sequences, even when under schematic control, is crucial for furthering the understanding of higher-level task sequences.

### Muscle Activation Sequences

Movements that involve multiple muscle groups can be characterized as sequences, as they require the temporal control of muscle activation and inhibition. For coordinated, cyclical movements that do not require persistent attention to execute (e.g., breathing, walking, pyloric rhythm) there are central pattern generators in subcortical structures, the spinal cord and the periphery to control these behaviors, and thus limit cognitive control and cortical involvement (Marder and Bucher, 2001). Often these behaviors are innate, or once learned, are not subject to extensive modification. Overlearned movements can be offloaded to extracortical structures as they become nearly reflexive, despite the fact that they may be composed of multiple complex steps in animals (Ito, 2000; Doyon et al., 2002) and humans (Toni et al., 1998; Swett et al., 2010). While it is possible for supervisory control to override the timing and expression of automatic behaviors (e.g., telling yourself to breathe), such nearly automatic sequences do not rely on higher cortical areas for expression and thus would fall under the purview of schematic control.

### Habitual Motor Sequences

Habits and addictive behaviors often involve the repetition of motor acts. Reward-action associations are represented by differential activity of regions throughout the brain, but particularly within the basal ganglia (Figure 1A). For example, the striatum is necessary for associating a particular action and reward, e.g., always press the right button for a reward (Berke et al., 2009). Stimulus-response associations represented in the striatum extend to entire sequences of actions that may become habitual. Studies in rodents have shown that neurons in the striatum mark the boundaries of action sequences (Jog et al., 1999; Barnes et al., 2005; Jin and Costa, 2010; Smith and Graybiel, 2013; Jin et al., 2014). This representation develops through learning (Jog et al., 1999; Barnes et al., 2005) and results in a sequence representation that does not include the specific, intermediate steps of the sequence (Jin et al., 2014). Studies in primates have also shown striatal activity at the boundaries of movement sequences (Fujii and Graybiel, 2003, 2005; Desrochers et al., 2015a). This striatal activity in primates also develops through learning, and activity at the end of the movement sequence may represent an integrated cost/benefit signal that can drive the acquisition of more efficient sequences (Desrochers et al., 2015a). Additionally, the basal ganglia play a critical role in the temporal control of movement sequences. Inactivation of the main motor output unit of the basal ganglia, the sensorimotor area of the globus pallidus internus, slowed the steps of a sequential out-and-back reach task, but did not interrupt the step order or completely inhibit the primates’ movement (Desmurget and Turner, 2010).

These studies suggest that motor sequence storage is not the primary role of the basal ganglia for well-learned, routinized actions, and that the basal ganglia likely serve as a gating mechanism for movement and competing action plans, i.e., play a role not only in schematic control, but also in supervisory control. Such supervision would require the evaluation of an entire series of actions, which neurons in the striatum have been shown to do (e.g., Desrochers et al., 2015a). Pharmacological inactivation of the caudate in primates during a double-step saccade task revealed the existence of competing motor plans. During the caudate inactivation, the subjects exhibited an increased number of averaged saccades, curved saccades and sequence errors (Bhutani et al., 2013). Although the concept of competing motor plans has been observed in cortical neural recordings and human behavioral tasks (Cisek and Kalaska, 2005; Gallivan et al., 2015) the extent to which this competition is observed and how the competing plans are chosen is still not well understood. Additionally, habit learning can induce strong links between steps, which can cause the completion of an earlier step to become the cue for a subsequent step in a series.

### Supervised Movement Sequences

**Figure 2:**
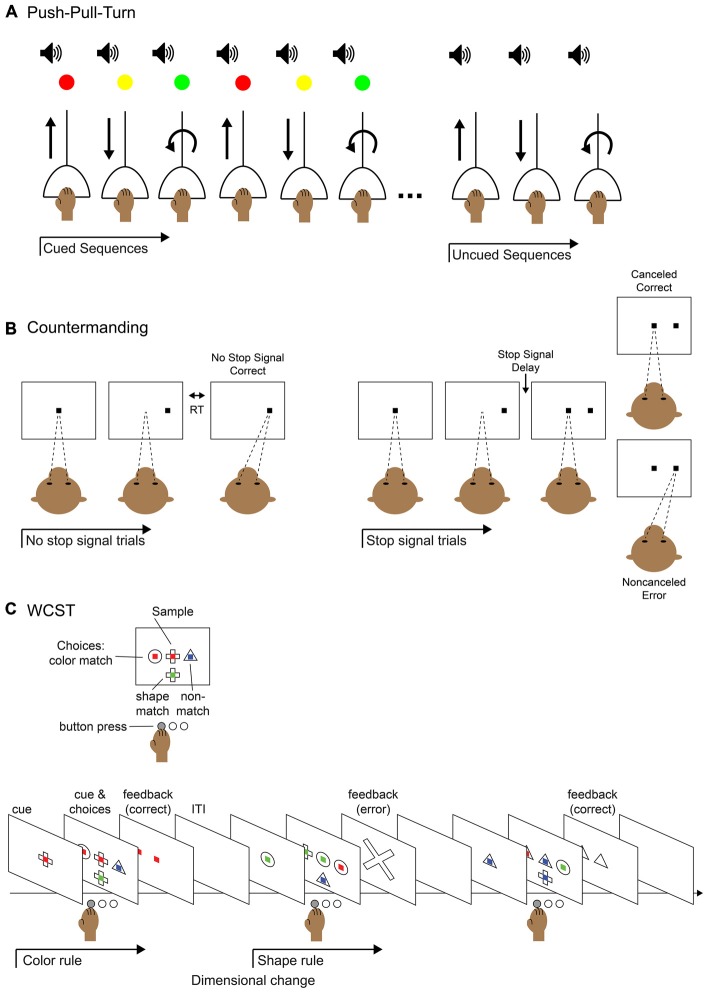
**Behavioral tasks for studying elements of control. (A)** Push-pull-turn task. The subject is instructed to complete a series of movements with an audio cue for movement timing and a light cue that indicates which movement to perform. After a few blocks of trials, the light cue is removed and the subject continues performing the remembered sequence of movements. **(B)** Saccade countermanding task (a.k.a. stop-signal task). The subject is instructed to hold fixation on a central fixation point until it is extinguished and make a saccade to a target that appears in the periphery; this is called a “no-stop” trial. On a fraction of trials, after the initial fixation point is extinguished and before the peripheral target appears, the fixation point reappears in the center. This is the “stop-signal” to abort the saccade and maintain fixation on the central fixation point. On a “stop trial,” maintaining fixation would be correct and executing the saccade to the periphery would be an error. The duration of the time before the fixation point reappears, the stop-signal delay, can be modulated to titrate the difficulty of the task: the longer the delay, the more difficult to it is to abort the saccade. **(C)** Wisconsin Card Sort Task (WCST). The subject is instructed to select the visual stimulus that matches the sample stimulus based on one of two rules: color match or shape match. The current rule is determined by trial and error and remains in operation until a dimension change. The subject presses a button to select the appropriate stimulus and is given feedback after the response.

As many sequential tasks are not composed of actions with rigid ordinal positions, and can happen on varying time scales, it is critical to study behaviors that allow for different ordering and combinations of movements, necessitating oversight by the supervisory control system. Various behavioral tasks have been used to study the execution of non-habitual motor sequences, including saccades (Zingale and Kowler, 1987; Petit et al., 1996; Grosbras et al., 2001; Isoda and Tanji, 2004), arm movements (Morasso, 1981; Wainscott et al., 2005; Overduin et al., 2008; Moisello et al., 2009; Panzer et al., 2009) and hand movements (Miyachi et al., 1997; Shima and Tanji, 2000). One particular task, the “push-pull-turn” task (Figure 2A), helped elucidate the role of the supplementary (SMA) and pre-supplementary (pre-SMA) motor areas (Figure 1A) in the control of sequential movements. Non-human primates learned to complete three different movements in different orders, initially with the aid of cues at each step. They were subsequently trained to perform the different motor sequences from memory, with only a single cue used to signify which sequence to execute. The investigators discovered three notable patterns of neural activity from single-unit recordings in pre-SMA and SMA: sequence-specific activity that varied during the first trial of each sequence type, position-specific activity that tracked the rank of the three movements, and interval-selective activity which varied depending on which movement had just been completed and which was next. In a separate experiment, the same investigators demonstrated that the inactivation of SMA interrupted the execution of a motor sequence, but not the execution of each individual movement (Shima and Tanji, 1998), as would be expected from an area involved in the supervision, but not direct execution of movements. Single-unit recordings in SMA and pre-SMA during an eight-stage sequence also showed activity related to the numerical ordering of the movement stages (Clower and Alexander, 1998). The neural coding of multiple facets (sequence, position, and interval) of movement sequences suggests mechanisms by which a supervisory controller may act (Figure 1B). Simultaneously, these studies provided a useful method for investigating how neuronal populations code for the phases and transitions of motor sequences.

Human and primate studies also indicate that activity in the frontal cortex plays an important role in the supervision of motor sequences. Imaging studies have demonstrated that neural activity varies depending on the type of sequence and stage of learning. When human subjects learned to complete multiple sets of key presses, both prefrontal cortex (PFC) and lateral premotor cortex activation increased during new sequence learning, while SMA activity increased during the execution of pre-learned sequences (Jenkins et al., 1994). In another study, interval and rank information were related to different levels of frontal cortical activity within the same network. Pre-SMA was more activated by interval information, while the SMA and frontal eye fields were activated more by rank-order information (Schubotz and von Cramon, 2001). On a finer spatial-temporal scale, subpopulations of neurons in the SMA, pre-SMA, dorsal lateral prefrontal cortex (DLPFC), and supplementary eye fields (SEF) exhibited rank-order activity (Berdyyeva and Olson, 2010). Neural recordings in anterior cingulate cortex (ACC) during a sequential trial-and-error problem solving task also showed activity related to rank-order (Procyk et al., 2000). These findings demonstrate that there is distributed processing across cortical areas during sequence expression. However, despite associations between particular areas and sequence task variables, it remains unclear how the responses in these cortical areas jointly represent sequence learning, intervals and rank information. The continued study of sequences of tasks would serve to demonstrate the different roles these areas play in tracking sequence expression.

In order to fully understand how the brain is able to monitor and complete the stages of a task sequence, it is important to decouple automatic, procedural tasks and tasks requiring supervisory control by considering the attribution of errors. For example, if you realize you had forgotten to add water to the coffee maker after turning it on, it would not make sense to throw the grounds out and restart from the beginning. Rather, it would be sensible to temporarily turn off the machine, add water, and continue, despite the misordered step. This monitoring of the higher-level goal, to make the coffee, allows for flexibility in how the task is achieved. This level of executive function requires interaction among error monitoring, attention and cognitive control circuits, which we will discuss in the following sections.

## Monitoring of Errors and Conflict

Many theories of executive control have emphasized the necessity of monitoring processes (e.g., Logan, 1985). Early work using event-related potentials (ERPs) described the error-related negativity (ERN) that is observed during error trials and is localized to medial frontal cortex (Gehring et al., 1993). Sequences of tasks require monitoring at both the higher-order sequence level and at each stage. There are at least two, non-exclusive alternatives for how higher-order monitoring could occur: (1) in a sustained fashion such that the sequence is constantly monitored against a reference set that determines how next to proceed; or (2) in a transient fashion at crucial choice points, such as the boundaries (beginning and end) of a sequence. There is strong evidence that online monitoring is key to handling perturbations of coordinated and goal-related movements (Todorov and Jordan, 2002; Scott, 2004; Diedrichsen et al., 2010), but it remains unknown whether a similar mechanism is used to monitor performance during a sequence of cognitive tasks. We propose that this monitoring is carried out by a supervisory controller that comprises a constellation of neural areas and that for real-world, naturalistic sequence tasks, this monitoring requires recurrent interactions between these areas in an active and dynamic way.

Few studies have directly examined transient vs. sustained dynamics of cognitive monitoring. One such study used a hybrid fMRI design during a task switching paradigm (Braver et al., 2003). The authors found evidence for transient activity in left lateral PFC and sustained activity, elevated throughout task performance with respect to baseline/no task, in right anterior PFC; both were modulated by trial-by-trial differences in response speed. These results provide initial evidence for both kinds of control dynamics (sustained and transient) and suggest that they are separable in the brain. Another study used a wide variety of identification, matching, search, and judgment tasks and found both transient and sustained dynamics in many different frontal-cortical areas, suggesting that different monitoring dynamics were not unique to PFC (Dosenbach et al., 2006). Because these tasks share many properties with sequence tasks, we will discuss examples of them and some of the commonly reported transient cortical dynamics that are associated with aspects of these tasks. In particular, we focus on two monitoring processes: error monitoring and conflict monitoring.

Several medial frontal cortical areas (Figure 1A) have been implicated in error monitoring by their selective response to errors. A common task used to study errors is the countermanding task (Figure 2B). In this task, the participant is instructed to make a movement to a cued peripheral target following a go signal. However, on a fraction of trials, rather than completing the cued movement the participant is presented a stop signal which instructs them to abort the execution of the planned movement. Monkeys and humans perform this task similarly (Emeric et al., 2007), and error responses in this task have been localized to the ACC using event related potentials (ERP) in humans (Godlove et al., 2011; Reinhart et al., 2012), as well as local field potential (LFP; Emeric et al., 2008) and single-unit (Ito et al., 2003) recordings in monkeys. Similarly, the SEF have been implicated in error monitoring in studies using human fMRI (Curtis et al., 2005), monkey LFP (Emeric et al., 2010) and single unit (Stuphorn et al., 2000; Schall et al., 2002) recordings in the countermanding task. The nearby SMA has also been implicated (Garavan et al., 2002; Scangos et al., 2013). A recent study using human intracerebral recording concludes that the SMA is the main locus of action monitoring because it shows responses during error trials before those of more rostral or pregenual ACC (pACC; Bonini et al., 2014). Further, responses in SMA were found without correlated responses in pACC, but not the opposite. This suggests a hierarchy within the medial frontal cortical monitoring network, where activity in the SMA precedes and influences activity in the pACC. However, we note that while pACC and postgenual ACC may have related functions, they are likely not the same. In general, naming conventions for the medial cortex surrounding the cingulate sulcus have not been consistent (for a review, see Procyk et al., 2014). For the purpose of this review, we will refer to postgenual ACC (sometimes dorsal ACC) as simply ACC and note pACC when applicable. It is likely that the division is not simple, and further investigation with more complex tasks will be necessary to more fully distinguish the roles of all these medial frontal cortical areas in error monitoring.

A concept related to error monitoring is conflict monitoring. Conflict monitoring allows for further engagement of cognitive control systems to resolve incompatibilities (e.g., respond to the color of the word “BLUE” when presented in a red font as in the Stroop task) as they arise, so that subjects can respond appropriately (Botvinick et al., 2001). In complex tasks, the ACC has been shown to respond to conflict in humans (Carter et al., 1999) and in the nearby pACC of monkeys (Amemori and Graybiel, 2012). Other studies have also suggested a more general role of ACC in outcome monitoring (for review, see Botvinick et al., 2004). A study varying the amount of conflict and the level of cognitive control/integration necessary for a response found that the ACC reliably responded to both conflict and subgoaling/integration demands (Badre and Wagner, 2004), again supporting a role of ACC beyond conflict monitoring. In this study, though it was not an explicit sequence, items were presented serially and knowledge of the serial order was required to make responses, further suggesting an evaluative role in sequences of tasks. ACC was also found to be one of the few areas that was activated at the initiation of many different kinds of cognitive tasks, and activation was sustained during task performance (Dosenbach et al., 2006). Therefore, in addition to the more specific/transient monitoring functions described in ACC, it is possible that the ACC also performs a more general monitoring function. However, it remains unclear how error or conflict monitoring processes function in true multistep tasks, as there is likely simultaneous monitoring of conflict with the higher-level goal governing the sequence and conflict within steps.

Clues as to how error and conflict monitoring processes may be carried out in sequences can be garnered from how those medial frontal cortical areas involved in monitoring—SMA, ACC, and SEF—respond during sequential tasks. Early studies using positron emission tomography (PET) imaging in humans showed that the SMA was activated for pre-learned sequences of saccades (Petit et al., 1996), and the ACC was associated with the acquisition of a implicit motor sequence (Grafton et al., 1998). The ACC did not show changes during sequence transfer or retrieval, suggesting that the ACC was critical for the rapid adaptation and monitoring necessary to detect and acquire a new sequence. The SMA and pre-SMA of monkeys has also been shown to respond to sequential movements in a large body of work (for review, see Tanji, 1994). Units recorded in the SMA have been shown to respond to the serial position in a sequence (Clower and Alexander, 1998; Isoda and Tanji, 2004) and the timing interval of sequential items (Shima and Tanji, 2000). Trial history also affects SEF activity during the countermanding task, which suggests that the SEF participates in planning of sequences in order to merge task history with task goals (Curtis et al., 2005). Neurons in the SEF and SMA respond to the serial order of items in a sequence (Berdyyeva and Olson, 2010) and units an the SEF can be selective to order within particular sequences (Lu et al., 2002). In an fMRI study in humans, triple-step saccades activated both SEF to trigger sequences and more generally, the ACC (Heide et al., 2001). These studies of motor sequences suggest that medial cortical monitoring areas may also participate in the supervision of motor sequences. However, these studies can only point at a parallelism by saying that the same areas shown to selectively respond to error or conflict also, during separate tasks, respond during sequences. Further research must be done before we can conclude that these areas code the presence of error or conflict truly simultaneously with the properties of sequences.

Few studies have directly examined error and conflict responses of medial frontal areas in the context of motor sequences. In an fMRI study in humans, participants performed a serial reaction time (RT) task with conflict produced by introducing responses that were out of sequence. The authors reported increased activation in conflict over no conflict trials in ACC (Ursu et al., 2009). ACC was also activated during errors, supporting a role of ACC in the evaluation and monitoring of sequences. The activity of monitoring areas does not always appear to scale simply, and may indeed interact with the control of sequences. In a pair of studies that illustrate this point, monkeys were required to touch targets in one of six sequences that were discovered by trial and error (Procyk et al., 2000). Task related neurons in the ACC coded the serial order of sequences, irrespective of kinematics. Some neurons preferred the search phase, when the monkey was actively trying to discover which of the six sequences to perform, while others preferred replication, when the monkey was repeating the discovered (correct) sequence. Subsequent work showed that this activity in the ACC was not just error monitoring, because the majority of the cells did not respond to error (Procyk and Joseph, 2001). These studies show that while monitoring regions can also encode elements of sequences, these coding properties are not necessarily simply additive and may interact to lead to novel representations, not yet well understood.

The interaction between monitoring and sequential control can be more closely examined through causal manipulations. There is limited evidence in this domain, but those studies that do exist strongly suggest that these medial frontal areas do not just monitor sequences, but perhaps actively control them. In humans, sequences of memory guided saccades were disrupted by lesions to the ACC (Gaymard et al., 1998). Lesions to SEF in humans also impaired the performance of memory-guided sequences of saccades (Gaymard et al., 1990, 1993; Heide et al., 1995). Microstimulation in the SEF of monkeys perturbed the order of saccades to two remembered locations, but did not seem to perturb the memory itself (Histed and Miller, 2006), and disrupted the ability of monkeys to select three targets in sequence (Berdyyeva and Olson, 2014). Similarly, another study in monkeys showed that the execution of motor sequences, but not individual movements, was disrupted by the inactivation of SMA (Shima and Tanji, 1998). All of these areas (ACC, SEF, and SMA) have also been shown to participate in monitoring as well, and the disruption of sequential performance when the functioning of these areas is perturbed again suggests that they play a supervisory control role in addition to monitoring sequences.

The studies discussed in the context of the functioning of monitoring brain areas thus far have used motor sequences. In a rare study of a sequence of three-item cued tasks (rather than motor sequences) followed by a long pause, the authors found significant activation in the ACC when a sequence of three tasks was initiated (Dreher and Berman, 2002). Consequently, the authors argued that the role of the ACC was not specifically about conflict, as the first item in the sequence would have no more or less conflict than the last item in the sequence, but more related to general alerting. Though the authors did not explicitly test for sustained dynamics in their study, the activation observed at the start of sequences could also reflect the heightened activity at the start of an epoch that required monitoring (Dosenbach et al., 2006). These studies suggest that monitoring areas may participate in the supervisory control of sequences of tasks along with motor sequences, but further research will need to be done to discern the exact nature of the involvement.

Perhaps the most suggestive evidence we have thus far that medial frontal cortical areas are involved in not only monitoring, but also sequencing, comes from a study that explicitly examined monitoring of an abstract (non-motor) sequence. In this study, human participants monitored serially presented letters for the presence or absence of a particular sequence or sequences of letters (Farooqui et al., 2012). Many areas in the fronto-parietal network showed greater activation for the detection of a sub- or end-goal target than intervening targets such as rostrolateral prefrontal cortex (RLPFC), ACC, and pre-SMA. Though the study did not explicitly report the significance of activity in those regions at each step in the sequence, plots of the activation in those regions of interest (ROIs) suggest that some, if not all, could also have significant activation levels at all steps in the sequence. Preliminary data from one other study shows neurons recorded in the PFC and hippocampus of rats respond during a sequence monitoring task (Quirk et al., 2014). This task requires participants to monitor a pre-learned sequence of either odors, in rats, or images, in humans, for an item that is out of sequence (Allen et al., 2014). Rats and humans showed similar behavior, suggesting that perhaps similar neural control mechanisms might be at work.

We have discussed three brain areas that are often associated with monitoring functions in the medial frontal cortex: ACC, SMA, and SEF. Activity in these three monitoring areas has been related to errors and conflict, but little is known about their direct involvement in the control of actions. Recent work in the ACC localizing feedback-related activation to individual participants’ specific motor map morphology in the same region may provide inspiration for future research on this topic (Amiez et al., 2013; Amiez and Petrides, 2014). Many studies suggest that these areas may function to exert control over sequences as responses to the ordering of stimuli and disruption of sequential performance are common findings with medial cortical recordings and manipulation. Together these studies suggest that the ACC, SMA and SEF are ideally situated to contribute to a supervisory role in task sequences, but very few studies have brought together investigation about monitoring and sequences, particularly on a more abstract task level. Though it is tempting to say that the monitoring and sequential control functions of medial frontal cortex are simply additive, it is most likely that there is an interaction between these functions and that each area has it’s own unique contribution to the process. Few studies have examined where these systems intersect and a small number have begun to distinguish the processing of the three areas discussed. Future work will be necessary to examine supervisory control and monitoring functions in the context of sequences of tasks.

## Attention: The Interplay Between Task Rules and Salience

The study of attention includes a vast body of literature; our interest here is to discuss the role of attention in the execution of sequential tasks. Specifically, the abstract “rules” that govern task performance must interact with lower-level task features, such as stimulus salience. Neurophysiology and imaging studies have demonstrated that attention correlates can be observed in many areas of cortex. Thus it seems more fruitful to consider shifts of attention in the context of circuits. Recent work suggests that shifting between cortical and subcortical circuits, inter-area synchrony and oscillations play a major role in control of attention (for a thorough review of oscillations and attention, see Baluch and Itti, 2011; Miller and Buschman, 2013). Each of these mechanisms has its own time course and the potential to uniquely contribute to the proper execution of sequential tasks.

Attention is generally characterized as having two distinct directional influences: top-down modulation (under supervisory control) and bottom-up modulation (which can activate schematic control). The features of a visual stimulus (e.g., brightness, contrast, color) can encourage orienting to that stimulus based on salience. For example, a background with very bright distractors can increase the time it takes to find an object of interest because the features of the distractors overwhelm the features of the target. During a sequential task, such bottom-up attentional drive could be either distracting (e.g., supporting the completion of steps in the wrong order) or enhance task performance by reinforcing sequence completion (e.g., decreasing possible options during the course of a task). Successful completion of a sequence of tasks relies on a continuous balance between the information channeled in from sensory cortices and top-down information about higher level goals.

Higher-level goals used by the supervisory control system are thought to be implemented by frontal cortical areas. The goals are thought to be represented by sustained activity in PFC during a task, and parietal areas might be the intersection of supervisory and schematic control systems (Asaad et al., 1998; Gill et al., 2000; Duncan, 2001; Wallis et al., 2001; Badre and Wagner, 2005; Sakai and Passingham, 2006). Frontal cortical neurons exhibit shorter latency than parietal areas carrying attention related signals (Buschman and Miller, 2007; Li et al., 2010), and microstimulation of the frontal eye fields can produce top-down modulation of area V4 in the ventral visual pathway (Moore and Armstrong, 2003), which demonstrates a mechanism for top-down attentional control. Inter-area coupling, including that between FEF and V4, and PFC and V4, has been shown to correlate with performance on visual attention tasks (Gregoriou et al., 2009, 2014). In addition, human neuroimaging has shown that superior parietal regions are involved in controlling shifts of attention, and has supported that such areas serve as an intersectional point between top-down and bottom-up attentional processes (Thakral and Slotnick, 2009; Greenberg et al., 2010). However the mechanism and site of interaction between attentional systems is still actively debated, as there are multiple sites where sequential and schematic control systems interact.

In the case of task sequences, top-down information can change from one step in the sequence to the next based on the current position within the sequence. The task goals might require orientation towards one feature of the stimulus during one phase, and a different feature in the next phase. The ability to change the focus of attention appropriately can be affected by factors such memory and trial timing. For example, memory can serve as an override of saliency. Memory-guided saccade sequences are less susceptible to distractors than cued saccade sequences (Gersch et al., 2009). Likewise, long-term memory can increase the sensitivity to the presence of a stimulus in particular spatial locations during visual search (Stokes et al., 2012). Evidence also supports that the rhythm of trial presentation is tracked in multiple areas, including fronto-cortical areas and auditory cortex (Cutanda et al., 2015; Konoike et al., 2015). These studies suggest that top-down attention is not a static process, but can adapt to the moment-to-moment changes in task demands while maintaining the over-arching goal.

Paradigms that involve task switching and different attentional networks have clarified the interaction of types of information (e.g., rules and bottom-up priming) and the roles of prefrontal and parietal areas in attentional shifts. One study decoupled top-down and bottom-up effects by asking people to maintain two separate mental counts, each associated with particular stimuli (Gehring et al., 2003). On each trial, participants either updated the same count as the previous trial (no-switch trial) or a different count (switch trial). No-switch trials facilitated faster RTs and shorter latency event-related potentials in frontal cortex, and this effect was exaggerated when the stimulus was also repeated. When the top-down (rule for which count to update) and bottom-up (stimulus viewed) components of the tasks aligned, attentional processes worked in synchrony. Another study directly investigated the effect of a PFC lesion on an attention task and found a behavioral deficit when the cue shifted rapidly across trials (Rossi et al., 2009). However, behavior was close to normal when the cue was constant across many trials and during a pop-out task with changing targets, which did not rely on top-down control. This suggests that the attentional systems can operate individually in certain tasks, although this independance may not hold for all paradigms. Ruthruff et al. (2001) proposed that task expectancy, defined as a top-down feature, affects the time to program an upcoming response, while task-recency, defined as a bottom-up attentional feature, affects the time to execute the response. They proposed that the two attentional systems jointly produce task readiness. This remains to be validated with neurophysiological evidence, but provides a testable hypothesis for the function of attentional systems in response preparation. Together, these studies suggest that both top-down and bottom-up attentional systems may contribute to the execution of sequential tasks, but direct evidence of the relative contributions of each attentional system through time in sequential tasks has not yet been demonstrated. It is likely that cognitive control mechanisms mediate the attentional systems described above, and thus we focus on this topic next.

## Flexible Adaptation for Goal-Directed Sequences

The elements of sequential control that we have discussed thus far: sequential movements, monitoring, and attention must ultimately be brought together to accomplish a sequence of tasks. Cognitive control is the ability to flexibly adapt behavior and select actions based on goals. This ability becomes particularly important when completing a sequence of tasks, as not only must the correct actions be selected, but they must be selected in an appropriate order, all the while maintaining the overall goal.

The PFC has been shown to be critical for these cognitive control functions and support the “rules” that govern goal-directed behavior in humans (Passingham and Rowe, 2002, for review, see Miller and Cohen, 2001), and in non-human primates (Wallis et al., 2001; Roy et al., 2010; Buschman et al., 2012; Rigotti et al., 2013, for review, see Fuster, 1993). The cognitive control of task sequences can be thought of as hierarchical in that multiple sub goals are created in the service of an overarching goal through time. Studies of non-sequential hierarchical control in humans have illustrated a caudal to rostral progression in the response of areas to progressively more abstract levels of the hierarchy (Koechlin et al., 2003; Badre and D’Esposito, 2007; Badre et al., 2009), that may be “gated” by the striatum (Badre and Frank, 2012). These studies suggest that the same frontal cortical areas may function similarly when the hierarchy is created by a sequence, rather than a static rule structure. In monkeys, neurons in the PFC were also found to be selective to the memory of a particular sequence of items (Warden and Miller, 2010), suggesting that the these monitoring and cognitive control functions of the PFC extend into the sequential realm.

A more anterior region, RLPFC has also been implicated in settings that have elements in common with sequential hierarchical control including: tracking and performing operations on items presented serially (Braver and Bongiolatti, 2002; Badre and Wagner, 2004; De Pisapia et al., 2012; Nee et al., 2013); performing multiple tasks simultaneously (Gilbert et al., 2006; Dreher et al., 2008); exploring, tracking and updating reward contingencies (Daw et al., 2006; Kovach et al., 2012); the highest level of a contingent rule structure (Badre and D’Esposito, 2007); and task switching (DiGirolamo et al., 2001; Kim et al., 2012). Many of these functions share aspects of monitoring superordinate goals to provide a top-down superordinate signal over the course of several trials (Braver and Bongiolatti, 2002; Badre and Wagner, 2004; Dreher et al., 2008; De Pisapia et al., 2012; Nee et al., 2013). Complementary findings have shown the time course of RLPFC activity to be sustained over many individual actions or choices (Koechlin et al., 1999, 2003; Braver et al., 2003). There are relatively few studies of RLPFC in animals because rodents do not have cortex that is homologous to RLPFC (Preuss, 1995) and techniques have been developed only recently to record from these areas in the non-human primate (Mitz et al., 2009). Existing work has implicated RLPFC in monkeys in feedback during set shifting (Tsujimoto et al., 2010, 2012), learning the value of behaviors (Boschin et al., 2015), and shifting attention (Caspari et al., 2015). These studies suggest that the RLPFC may function similarly in the monkey and in the human, but none of these studies in monkeys or humans explicitly examine the functioning of RLPFC during sequential task control.

Many paradigms have been used to examine the flexible capabilities of frontal cortex in cognitive control. We will briefly highlight two, task switching and the Wisconsin Card Sorting Test (WCST), because of the adaptability of these paradigms to examine sequential task control. Both tasks already begin to query elements necessary for sequential control because it is only in the context of the previous task that the current task is a switch in task or “rule”. In task switching, the increased time that it takes to go from one task to another is used as a marker for the engagement of cognitive control mechanisms in both humans (Rogers and Monsell, 1995; Ruthruff et al., 2001), and monkeys (Stoet and Snyder, 2003a,b, 2009; Caselli and Chelazzi, 2011). Task switching studies using fMRI in humans have revealed the activation of a wide array of areas in the frontal-parietal network such as RLPFC, PFC, and medial frontal cortex (Dove et al., 2000; Sohn et al., 2000; DiGirolamo et al., 2001; Braver et al., 2003; Kim et al., 2012; Schuck et al., 2015, for review, see Ruge et al., 2013). In monkeys, neurons that respond to particular strategies or the shift in strategies have been recorded in the PFC (Genovesio et al., 2005, 2008) and RLPFC (Tsujimoto et al., 2010, 2012). While none of these studies explicitly studied sequences of tasks, the areas that were found to be activated in task switching are also often implicated in monitoring, attention, and cognitive control, suggesting that the combination of these elements necessary for the execution of task sequences may have a neural substrate in one or more of these brain regions.

The WCST requires shifting rules or strategies where the switches are learned by trial and error and are not signaled or predictable, and thus require tracking the rules through time. Participants must “sort” the cards according to one dimension of the stimuli presented, such as color or shape. In adaptations of this task, the equivalent is deciding which dimension is currently relevant to match to the current stimulus (Figure 2C). This paradigm is different than instructed task switching, but seems to engage many of the same regions. Human lesion and imaging studies have shown the involvement of PFC in shifting or feedback (Milner, 1963; Berman et al., 1995; Nagahama et al., 1996; Monchi et al., 2001; Nakahara et al., 2002) along with ACC during error trials (Lie et al., 2006). Monkeys can learn analogs of the WCST (Mansouri and Tanaka, 2003; Moore et al., 2005). As in humans, studies in monkeys have shown the PFC is involved in maintaining the current rule and monitoring performance (Mansouri et al., 2006; Buckley et al., 2009; Moore et al., 2009), the RLPFC is involved in adapting performance according to the history of conflict (Mansouri et al., 2015), and the ACC is implicated in evaluating performance (Buckley et al., 2009; Moore et al., 2009; Kuwabara et al., 2014). It is often assumed that there is functional homology between brain areas involved in performing similar tasks in monkeys and humans. In a rare study directly comparing activations found in fMRI of monkeys and humans performing the WCST they found that set shifting activity was localized to the PFC of both species (Nakahara et al., 2002). These findings are important because there is no guarantee with the limited scope that recording electrodes have that they will capture the activity of those neurons most active/important for the task. Together these studies of task and set shifting implicate areas in the frontal lobes that are commonly associated with more general cognitive control. Understanding how exactly each of these areas is involved when any switch of set or task is executed within a sequence will require studying task sequences directly.

There are many unique demands when executing tasks sequentially, as evidenced by the fact that patients with frontal lobe damage are often unable to perform everyday task sequences on their own, despite the ability to perform normally on other tests of executive function (Eslinger and Damasio, 1985; Shallice and Burgess, 1991). For example, one patient was unable to perform complex sequences required daily living, yet excelled at the WCST. The patient could complete tasks towards specified goals only when the tasks and goals were repeatedly presented externally. The patient also seemed unable to trigger the automatic programs necessary for self-care (e.g., feeding). However, he was capable of initiating single movements and did not have any kind of movement deficit. This then again highlights several components of task sequences that are not captured by classic tests of executive function. Task sequences require flexible allocation of resources and time to complete multiple sequential goals, and are often unguided by external cues. Therefore, successful completion of a task sequence requires organization, internal monitoring, and the interaction between neural circuits are involved in schematic and supervised control. To study these elements that are unique to task sequences, it is then important to push an experimental paradigm beyond classic tests of executive function. With the large body of literature supporting task switching effects under many conditions, switching tasks in sequences is an ideal paradigm to study this kind of sequential control. When sequences of tasks are performed in everyday life, it most closely resembles a hierarchical task switching behavior, as we maintain an overarching goal while accomplishing, and switching between, many subtasks. It also has been shown that switch costs are robust to how much preparation a participant has to switch tasks, even when which task they are to complete next is completely memory guided (Sohn and Carlson, 2000).

Behavioral evidence for the hierarchical control of task sequences came from a study that asked participants to perform simple stimulus categorization tasks according to a remembered sequence (e.g., color, shape, shape, color; Schneider and Logan, 2006). They showed increased RT costs at the first item in the sequence, over and above costs of task switching alone. This provided evidence for the hierarchical control of task sequences because in the absence of the execution of a sequence first position RT’s would not be elevated.

Despite their ubiquity in everyday life, we know little about how the brain controls task sequences (Koechlin et al., 2000; Koechlin and Jubault, 2006; Farooqui et al., 2012; Desrochers et al., 2015b). In Farooqui et al. (2012), participants monitored a stream of individual letters for targets from pre-specified sequences of different lengths. The primary result was that a broad network of frontal and parietal areas, including RLPFC, PFC, ACC and pre-SMA showed increased activation at the sequence termination. This provides evidence for these areas participating in the monitoring of abstract sequences, but the task did not require selecting a new task depending on sequence position (local task switching). Rather, the task level change was always at the sequence boundary. Therefore, the question of how these areas participate in the performance of task sequences is left open.

Another study of sequential control in humans asked participants to perform a sequence of choice RT tasks vs. a simple motor sequence during fMRI (Koechlin and Jubault, 2006). In this study, the task sequence was performed only once, and the initiation and termination were cued externally. They found phasic activation at the initiation and termination of the entire sequence of tasks in the inferior frontal gyrus (BA 45), and activation related to the initiation and termination of motor sequences more posteriorly in inferior frontal gyrus (BA 44) and in the pre-SMA. This suggests a separation of those areas engaged in the performance of task sequences from those involved in motor sequences that appears to support the notion that more abstract constructs are represented more anteriorly in the brain.

**Figure 3:**
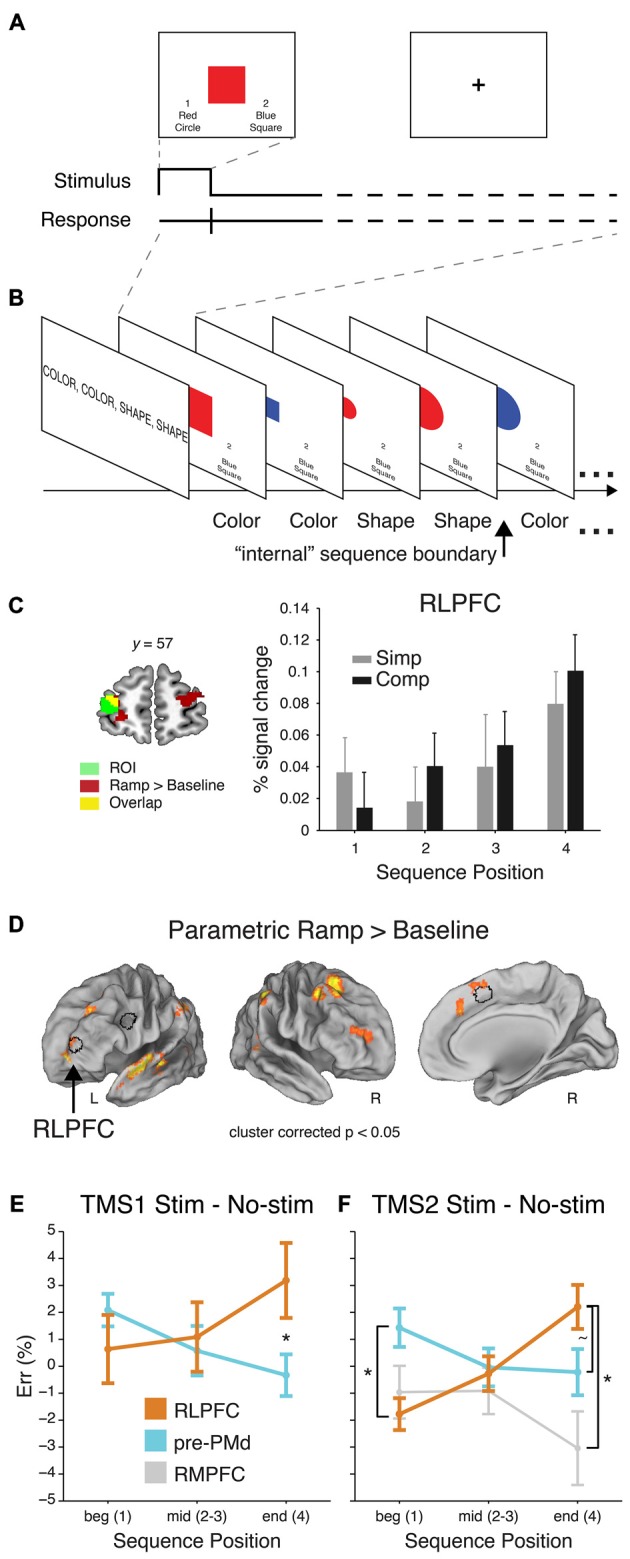
**Sequential control task, adapted from Desrochers et al. (2015b). (A)** Example trial. **(B)** Partial example block with the task that should be executed on each trial (as remembered from the instruction screen). **(C)** Left: RLPFC ROI. Right: Mean percent signal change (+SEM) from the peak event-related response (at 6 s) of the voxels included the RLPFC ROI. **(D)** Voxelwise contrast of the Parametric Ramp regressors over baseline (extent threshold 172 voxels, note lateral views rotated ~50°). Outline of the RLPFC, pre-PMd, and SMA/pre-SMA ROIs used in the study in black. **(E)** Mean difference in ER (±SEM) due to stimulation at peak SOA for RLPFC and for pre-PMd in TMS1. ER differences shown over the course of sequences: beginning (Position 1), middle (Positions 2 and 3), and end (Position 4). Asterisk indicates significant difference in the effect of stimulation at Position 4 (*F*_1,32_ = 6.7, *P* < 0.01). **(F)** Same as **(E)** but for TMS2. Asterisk at Position 1 indicates a reliable difference between RLPFC and pre-PMd (*F*_1,28_ = 6.2, *P* < 0.02). At Position 4, tilde indicates a marginal difference between RLPFC and pre-PMd (*F*_1,28_ = 2.9, *P* < 0.1), and asterisk indicates a reliable difference between RLPFC and rostromedial prefrontal cortex (RMPFC; *F*_1,14_ = 4.4, *P* < 0.05).

Based on previous behavioral work on hierarchical task sequences (Schneider and Logan, 2006), a recent fMRI and transcranial magnetic stimulation (TMS) study asked human participants to perform remembered sequences of tasks while undergoing fMRI scanning or TMS (Desrochers et al., 2015b). This study captured aspects of sequential task behavior that previous studies did not: participants had to both perform a task at each position in the sequence, and the initiation and termination of each sequence was internally monitored. The tasks were to make color and shape judgments of simple stimuli (Figure 3A). On each block of trials, participants were instructed to perform the tasks in a 4-item sequence, e.g., color-color-shape-shape, and they repeated this sequence, without external cues regarding the position in the sequence, for the duration of the block (Figure 3B).

In this context, the authors found that in the frontal cortex, the RLPFC, PFC, pre-SMA, and medial frontal cortex showed activity that gradually increased through the four-item sequence of tasks, and then reset at each new beginning (Figures 3C,D). Other areas in the frontal cortex, such as predorsal premotor cortex (pre-PMd) did show responses to other elements of this sequential task, but did not show the ramping pattern of activation and thus dissociated from RLPFC. Given the extent that RLPFC has been implicated in supervisory control functions, the authors then sought to determine if the ramping pattern of activation found in the RLPFC was indeed necessary for sequential task control and what the function of this activity might be. As a causal manipulation, TMS was applied during the same sequential task. The authors showed, in two separate experiments, that the RLPFC and associated network was necessary for the supervisory control of task sequences because single-pulse TMS caused an increase in the number of errors induced as the sequence progressed (Figures 3E,F). These effects mirrored the ramping pattern observed in fMRI (Figure 3C). The effects in RLPFC also dissociated from the effects of stimulation in the pre-PMd and a second control region, the rostromedial prefrontal cortex (RMPFC). These results suggest that the RLPFC is a key node in the supervisory control network for task sequences, and that its involvement is increasingly necessary as sequences progress and uncertainty may build up as to the current position within the sequence (Figure 1C). Previous studies of sequential control did not report this kind of ramping dynamic within the sequence (Koechlin and Jubault, 2006; Farooqui et al., 2012), suggesting that it is under these more naturalist conditions were participants must remember and monitor the sequence of tasks to be performed without external cues that these novel dynamics are revealed.

These few studies only scratch the surface of understanding sequential task control. Many questions remain as to the relative contributions of each area, how all the areas implicated in sequential control interact, and the underlying cellular mechanisms. It is in this realm that studies of nonhuman primates can be particularly informative; however, studies of sequential task control in these animals are even more rare than they are in the human. The neural mechanism underlying the ramping dynamics observed in humans may resemble the neural activity profiles that have been found in action sequences. Many regions such as the DLPFC, supplementary motor area (SMA), pre-SMA, and SEF have neurons that show selectivity to the serial position in action sequences (Niki and Watanabe, 1979; Clower and Alexander, 1998; Averbeck et al., 2003; Ryou and Wilson, 2004; Mushiake et al., 2006; Averbeck and Lee, 2007; Berdyyeva and Olson, 2010). Although these neural responses tend to be phasic at one position, some neurons may code positions later in the sequence with larger responses than earlier ones, thus producing the appearance of a ramp across the population (Averbeck et al., 2003; Berdyyeva and Olson, 2010). Examples of individual neurons that show ramping dynamics have also been found in the ACC and PFC (Niki and Watanabe, 1979). These cortical dynamics may interact with neuromodulatory mechanisms in the striatum, as the dopamine content of the striatum has been shown to ramp up as rats progress towards a goal (Howe et al., 2013). It has been suggested that two systems exist in parallel that use more spatial task-based coordinates or motor coordinates for sequential control, both containing loops through the basal ganglia and frontal cortex (Hikosaka et al., 1999) and that the neural constituents of sequential monitoring may be hierarchically organized themselves (Sigala et al., 2008). Further study of task sequences specifically will be necessary to illuminate these hypotheses.

In order to bridge the investigation of the neural basis of sequential task control between monkeys and humans, it is crucial to develop sequential task paradigms that can be performed by both species. It is not sufficient to assume similar tasks will be controlled by similar underlying neural mechanisms, and there are likely several levels of interactions between the neural responses in relatively simple tasks, and task sequences. As an illustrative example, in a rare study of monkeys performing sequences of tasks separated by long intervals, standard task switch effects were not observed (Avdagic et al., 2014). Techniques such as the use of fMRI in monkeys will also be key to establish functional homology between monkeys and humans, as it will allow the direct comparison of the activations present in each species (when used with the same task).

**Figure 4:**
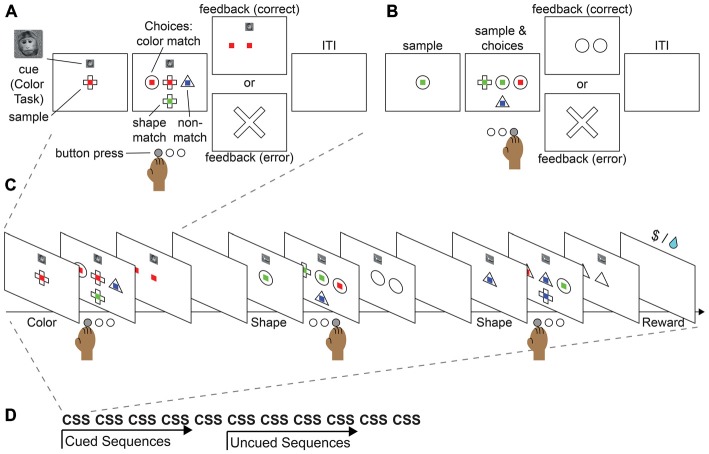
**Proposed monkey task sequence paradigm.** This task merges the features of the push-pull-turn task, the countermanding task, and WCST into a single sequential paradigm. **(A)** Example cued trial. **(B)** Example non-cued trial. **(C)** Example sequence. **(D)** Example block.

We provide here an example of a task that could be used to study sequential task control in monkeys and in humans. The paradigm merges the key aspects of the well-studied push-pull-turn, countermanding, and WCST tasks into a sequential control task. In this task, participants would be asked to match a central sample stimulus to one of three choice stimuli, according to their color or shape (Figure 4A). During initial training, an image displayed above the central sample stimulus would serve as a cue for the shape or color rule. In each block of trials, participants would repeat a short sequence of cued judgments (e.g., color, shape, shape; Figure 4C). After significant training, subjects would begin by performing a three-task sequence, and after completion of five cued sequences, would continue performing the same sequence, but without cues (Figure 4B) in order to complete a block of trials (Figure 4D). The design builds on the push-pull-turn task where a sequence of arm movements is first instructed, and then executed from memory (Figure 2A; Shima and Tanji, 2000). The important distinction between the push-pull-turn task and the current sequential task is that the sequence is not composed of individual movements, but is composed of the tasks to be completed (e.g., shape, shape, color) and completely removed from a motor sequence. This sequential task also builds on the capability of monkeys to flexibly adjust the current task or rule, and choose the appropriate stimulus dimensions on which to base a decision as in the WCST (Figure 2C; Nakahara et al., 2002). Together, these elements combine the monitoring, attention, and cognitive control requirements of task sequences into a paradigm that both monkeys and humans can perform, paving the way for future work.

## Conclusion

Despite the relative ease with which we complete sequences of tasks in our daily lives, they are incredibly complex and require the proper functioning of many systems in concert for their successful completion. We have discussed in this review the work in motor sequences that has provided a foundation for task sequences, and some of the major components of task sequences: monitoring, attention, and cognitive control. Very few individual studies or task paradigms bring together all of these components to study task sequences as a whole. Though often times it may be assumed that these neural systems may work similarly under sequential conditions as under non-sequential conditions, it is critical to test these assumptions to develop a direct understanding of task sequences themselves. This understanding is then important to address gaps in our understanding of how disorders that involve sequences such as Parkinson’s disease, Huntington’s disease, obsessive compulsive disorder, and perhaps even attention deficit disorder occur and may be treated. Simultaneously, an understanding of task sequences is important for aiding the large numbers of patients that have some form of frontal dysfunction and are unable to live independently.

We have proposed a framework that separates the control of sequences into schematic and supervisory control. The schematic controller selects sequences of movements that are more procedural and can be executed as a single unit. For example, many muscle activation sequences do not require specific attention in order to execute. While habitual motor sequences can also be executed as a unit and may be selected by the basal ganglia as part of the schematic control network, evidence suggests that the basal ganglia also take on more of a supervisory role for habitual actions, and play a central role in the formation and evaluation of these sequences. The supervisory control system is responsible for monitoring, handling any exceptions that arise, and keeping track of a higher-level goal. We have provided evidence that medial cortical areas implicated in monitoring functions may perform similar functions in sequences of tasks in the service of the supervisory controller. Attention harnesses the schematic controller in the form of bottom-up primary-sensory mechanisms that are executed without conscious regulation. Top-down attentional mechanisms are at work when frontal cortical brain areas bias the activity of downstream regions to accomplish a particular goal under supervisory control.

When one has to flexibly pursue goals that may change through time, as in task sequences, the role of flexible supervisory control becomes more pronounced. Generally these flexible control functions have been assigned to rostral frontal cortical areas in non-sequential tasks where the maintenance or flexible switching among abstract rules for action is necessary. Studies of sequential motor tasks have similarly suggested that these regions track the variables necessary for the tracking and control of elements of sequences, and the sequences as a whole. However, there are few studies that have examined the most abstract level of the supervisory controller—the control of sequences of tasks.

The few studies that have examined sequential task control start to give evidence of how monitoring, attention, and cognitive control may come together, but in novel ways. For example, it had been previously established that the RLPFC was selectively involved in the highest level of abstraction when completing complex tasks and could be activated in a sustained manner; however, the ramping dynamics observed through the steps in a sequence of tasks had not been observed prior to participants actually being asked to complete such a task sequence (Desrochers et al., 2015b). Thus, while the areas in the frontal cortex and striatum may all play their respective roles that are not dramatically different in sequential tasks from the functions they are associated with in non-sequential tasks or motor sequences, the dynamics of their functioning and how they connect with other areas during sequential tasks is largely uncharted territory. Further study is necessary to directly observe and manipulate the neural circuitry in sequential tasks, and tasks such as the one we have proposed that are capable of being performed by both monkeys and humans will provide a crucial bridge in understanding between the mechanisms and the actions of people.

## Author Contributions

All authors developed the topic for the manuscript. TMD and DCB completed the initial draft of the manuscript. All authors edited the manuscript.

## Conflict of Interest Statement

The authors declare that the research was conducted in the absence of any commercial or financial relationships that could be construed as a potential conflict of interest.
